# Probing the *Rhipicephalus bursa* Sialomes in Potential Anti-Tick Vaccine Candidates: A Reverse Vaccinology Approach

**DOI:** 10.3390/biomedicines9040363

**Published:** 2021-03-31

**Authors:** Joana Couto, Gonçalo Seixas, Christian Stutzer, Nicholas A. Olivier, Christine Maritz-Olivier, Sandra Antunes, Ana Domingos

**Affiliations:** 1 Instituto de Higiene e Medicina Tropical, Universidade Nova de Lisboa, Rua da Junqueira, 100, 1349-008 Lisboa, Portugal; gseixas@ihmt.unl.pt (G.S.); adomingos@ihmt.unl.pt (A.D.); 2 Global Health and Tropical Medicine, Instituto de Higiene e Medicina Tropical, Universidade Nova de Lisboa (GHTM-IHMT-UNL), Rua da Junqueira, 100, 1349-008 Lisboa, Portugal; 3 Division of Genetics, Department of Biochemistry, Genetics and Microbiology, Faculty of Natural and Agricultural Sciences, University of Pretoria, Pretoria 0002, South Africa; christian.stutzer@gmail.com (C.S.); christine.maritz@up.ac.za (C.M.-O.); 4 Department of Plant and Soil Sciences, University of Pretoria, Pretoria 0002, South Africa; nicky.olivier@fabi.up.ac.za; 5 Forestry and Agricultural Biotechnology Institute (FABI), University of Pretoria, Pretoria 0002, South Africa

**Keywords:** tick, vaccine, sialotranscriptome, reverse vaccinology, immunoinformatics

## Abstract

In the wake of the ‘omics’ explosion of data, reverse vaccinology approaches are being applied more readily as an alternative for the discovery of candidates for next generation diagnostics and vaccines. Promising protective antigens for the control of ticks and tick-borne diseases can be discovered by mining available omics data for immunogenic epitopes. The present study aims to explore the previously obtained *Rhipicephalus bursa* sialotranscriptome during both feeding and *Babesia* infection, to select antigenic targets that are either membrane-associated or a secreted protein, as well as unique to the ectoparasite and not present in the mammalian host. Further, they should be capable of stimulating T and B cells for a potential robust immune response, and be non-allergenic or toxic to the host. From the *R. bursa* transcriptome, 5706 and 3025 proteins were identified as belonging to the surfaceome and secretome, respectively. Following a reverse genetics immunoinformatics pipeline, nine preferred candidates, consisting of one transmembrane-related and eight secreted proteins, were identified. These candidates showed a higher predicted antigenicity than the Bm86 antigen, with no homology to mammalian hosts and exposed regions. Only four were functionally annotated and selected for further in silico analysis, which examined their protein structure, surface accessibility, flexibility, hydrophobicity, and putative linear B and T-cell epitopes. Regions with overlapping coincident epitopes groups (CEGs) were evaluated to select peptides that were further analyzed for their physicochemical characteristics, potential allergenicity, toxicity, solubility, and potential propensity for crystallization. Following these procedures, a set of three peptides from the three *R. bursa* proteins were selected. In silico results indicate that the designed epitopes could stimulate a protective and long-lasting immune response against those tick proteins, reflecting its potential as anti-tick vaccines. The immunogenicity of these peptides was evaluated in a pilot immunization study followed by tick feeding to evaluate its impact on tick behavior and pathogen transmission. Combining in silico methods with in vivo immunogenicity evaluation enabled the screening of vaccine candidates prior to expensive infestation studies on the definitive ovine host animals.

## 1. Introduction

Tick and tick-borne diseases are an increasing threat for both human and animal health [1]. The multi-host hard tick species, *Rhipicephalus bursa*, has a wide distribution throughout the Mediterranean basin and transmits several pathogens of economic importance in ungulates (i.e., cattle, sheep, and goats) from several genera, including *Babesia*, *Anaplasma*, *Theileria*, *Rickettsia,* and *Coxiella* [2]. Recently, *R. bursa* has been implicated in the transmission of several zoonotic pathogenic agents highlighting its impact in human health [3]. This tick is the primary vector of *Babesia ovis*, a highly pathogenic hemoparasite in small ruminants, recognized for having an important socioeconomic impact, primarily in low income countries, related with production losses and costs of the animal treatment [4]. Moreover, *B. ovis* is present in all developmental stages of *R. bursa* species since it has the capacity for transovarial and transstadial transmission [5].

The (re)emergence of ticks and tick-borne diseases and the lack of safer and more effective control strategies have reinvigorated research efforts by the scientific community to explore ways to control ticks and, subsequently, their associated diseases. Biological control [6], acaricides [7,8], resistant production breeds [9], and vaccines [10,11,12] are being proposed and readily tested for tick population control. Vaccines are one of the most environmentally friendly pharmaceutical products [13], as well as effective prophylactic treatment [14], used in infectious disease control. In tick research, the development and commercialization of novel vaccines [15] have been hindered by different factors [16,17] such as the lack of knowledge regarding tick-host interactions and tick biology. Particularly, transmission-blocking vaccines are considered very attractive tools for vector-borne disease control since they can affect the vector’s biology and behavior, thereby interfering with its capacity to transmit diseases [18,19,20]. Traditionally, vaccines confer protection by stimulating a humoral response mediated by antibodies [21]. These antibodies are involved in recognition and binding of the foreign antigen resulting in neutralization, agglutination, precipitation, as well as complement activation using chemoattractants to facilitate inflammation [22].

To date only one subunit anti-tick vaccine has been commercialized, based on a surface exposed 89 kDa glycosylphosphatidylinositol (GPI)-linked glycoprotein, Bm86, originally found in *Rhipicephalus microplus* midgut tissues [23,24]. Vaccination with this antigen can induce a protective immune response mediated mainly by host humoral response and the complement system, damaging the tick midgut wall and leading to a decrease in tick survival and diminished capacity to produce viable progeny [25,26,27]; however, with a efficacy depending on the tick species and strains [11].

Thus, a panoply of targets must be studied and tested to increase the current antigen repertoire for use in novel anti-tick vaccines and improve their efficiency. Immunoinformatics-based approaches have been recently applied to catalogue potential protective antigens, reducing cost and time in anti-tick vaccine development [25,28,29,30]. Reverse vaccinology (RV) is an approach exploring available omics data and in silico tools to select a great amount of predicted antigenic proteins potentially capable of inducing a protective immune response in vivo [21,31,32,33,34]. A combination of such techniques is steadily being implemented to develop novel and effective vaccines against several infectious diseases, including parasitosis [35,36,37,38,39,40]. These approaches have been already used on tissue-specific tick omics data in the pursuit of potential protective candidates [26,30,41,42,43]. Immunoinformatics focusing tick sialomics data are of particular interest since tick secreted salivary proteins can closely influence the host immune response at the vector feeding site, as well as enable pathogen dissemination and multiplication inside the host [44,45,46,47,48]. Even though functional redundancy is expected in tick salivary gland proteins [49], transcriptomic and proteomic studies of this tissue represents a collection of pharmaco-active molecules with therapeutic exploitation potential [48]. Moreover, peptide and multi-epitope constructs can be designed to synergize the impact of recognizing multiple antigens [50] and overcoming functional redundancy, as well as minimizing the side-effects caused by the immunization of an entire protein [51].

Regarding topology, proteins that contain extracellularly exposed portions on the cell outer membranes (surfaceome) or that are secreted into the extracellular space (secretome) are considered suitable antigens for vaccine development due to better accessibility to the immune system [26,32], as opposed to cytoplasmic proteins that can rather be considered for small molecule drug development [26,32,52]. Targeting a membrane-related protein may also interfere with the tick capacity to transmit or acquire pathogens if produced antibodies directly block the parasite from crossing the midgut or salivary glands barriers, as observed in other vectors, such as mosquitoes [18,53]. The tick secretome represents a chemical pool, critical for tick feeding and life cycle, as well as pathogen transmission. Thus, tick salivary secretions could be the core for the development of novel therapeutics for host disorders [48,54] or anti-tick and transmission blocking vaccines [55] as in the case of Salp15 and *Borrelia burgdorferi* transmission by *Ixodes* ticks [56].

Therefore, this study aims to scrutinize available high-throughput omics data, using a RV approach, focusing on the *Rhipicephalus bursa*-*Babesia ovis* (vector-pathogen) interface in order to identify antigenic peptides from tick sialoproteins that could be promising candidates for future vaccination trials. For this, computational methods have been combined to predict B and T cell epitopes, as well as its topology, hydrophobicity, polarity, solubility, and other physicochemical aspects [57], in order to select candidates comprising all the requirements for a suitable vaccine or even for disease diagnosis and disease therapy [58]. Such exposed antigens could become targets for peptide-based therapeutics if they present high antigenicity and no toxicity to the host.

## 2. Material and Methods

### 2.1. Rhipicephalus bursa Sialotranscriptomes: New Assembly

The data analyzed was obtained previously by Antunes et al. [59]. Briefly, *R. bursa* female ticks were obtained under different conditions: uninfected-unfed ticks, uninfected-fed ticks, and *B. ovis* infected-fed ticks. Salivary glands were isolated, RNA extracted, and two replicates *per* condition were used for RNA sequencing in a HiSeq 2500 sequencer (Illumina, CA, USA) after quality assessment and library construction. Cluster generation was performed, followed by 2 × 100 cycle sequencing reads separated by a paired-end turnaround. The raw fastq files deposited at the National Center for Biotechnology Information (NCBI) under the accession numbers SRR4428986, SRR4428987 and SRR4428988 [59] were re-analyzed under the present study. For this, sequence reads were quality filtered and trimmed using Trimmomatic [60] and the transcriptomes reassembled using Trinity [61]. To evaluate the completeness of the assemblies, BUSCO [62] analyses were performed using the Arthropoda dataset as a reference.

### 2.2. In Silico Characterization of Rhipicephalus bursa Protein Coding Sequences

**Figure 1:**
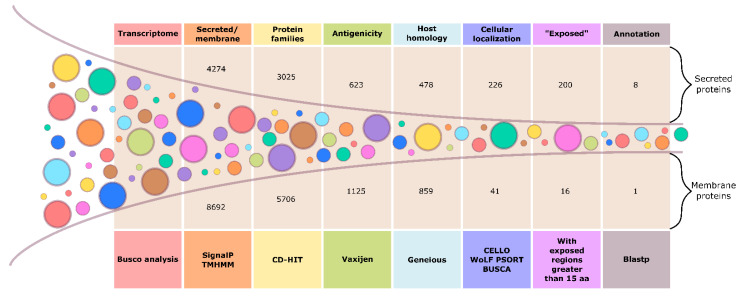
Graphical depiction of the reverse vaccinology (RV)-based methodology used for antigen screening. From the “fed and infected” *R. bursa* sialotranscriptome dataset, the secretome (secreted proteins) and the surfaceome (membrane proteins) were differentiated using SignalP and TMHMM (software package, standalone installation or online server, current v.2.0.). Several filters were applied using different programs to filter promising targets.

A filtering process was performed using various bioinformatics tools to identify protein coding open-reading frames, topological features, antigenic regions, and annotate potential candidates. See Figure 1 for the experimental outline.

Open reading frames (ORF) in transcriptome contigs were predicted using TransDecoder [62]. InterProScan [63] was used to classify the predicted protein sequences in terms of signal peptide and transmembrane regions with SignalP (v. 4.0) [64] and TMHMM (v. 2.0) [65,66], respectively. Redundant (or highly similar; identity >90%) and closely related protein families were analyzed with CD-HIT v4.8.1 [67], and only the representative sequences were used in subsequent analyses.

The online server Vaxijen (v. 2.0) was used to select the antigenic proteins [68]. Based on the immunogenicity of the only commercially available anti-tick vaccine, Bm86, a threshold of 0.7 was applied [30].

Then, to select antigens that differ from possible vertebrates used in future vaccination trials, the Geneious R8.1 software was used to search for homology between the *R. bursa* dataset and the mammalian hosts amino acid databases (*Mus musculus*, *Oryctolagus cuniculus* and *Ovis aries*).

Next, CELLO (v. 2.5) [69], WoLF PSORT [70], and BUSCA [70] online servers were used to select the surfaceome and secretome using the names “plasma membrane” and “extracellular” as filters. SignalP (v. 5.0) [71], big-PI [72], GPI-SOM [73], Phobius [74], TMHMM (v. 2.0), CCTOP (v. 1.0) [75], and SACS TMHMM [75] programs were used for the selection of membrane-related antigens with exposed regions (“outside” or “non-cytoplasmatic”, with regions greater than 15 a.a.) without signal peptide neither glycosylphosphatidylinositol (GPI)-anchor in all the in silico results.

For the selection of secreted antigens, the same approach was used including proteins with signal peptide but no GPI-anchor in all the in silico results.

Finally, the selected *R. bursa* proteins were functionally annotated by BLASTp (https://blast.ncbi.nlm.nih.gov/Blast.cgi?PAGE=Proteins, accessed on 30 October 2019) against the NCBI non-redundant (nr) and Arthropoda (6656) databases using the PAM70 matrix (E value < 1×10^−1^, coverage: 50–100%, identity: 50–100%). Only the proteins functionally annotated were further used in the ensuing analysis.

## 3. ORFs and Proteomics

The occurrence of the predicted ORFs from the transcriptomes in the previously published *R. bursa* sialoproteomic data was assessed. The proteomic data was obtained previously by Couto et al. [76]. Briefly, four groups of ticks were generated considering the conditions of uninfected unfed, uninfected fed, infected unfed, and infected fed, and salivary glands from each were dissected for protein extraction. Protein extracts were precipitated and digested, until the peptides were desalted; samples were analyzed via reverse phase liquid chromatography coupled online with mass spectrometry (RP-LC-MS/MS) using an Ekspert nLC 415 system combined to a 6600 TripleTOF^®^ mass spectrometer (AB SCIEX^®^, MA, USA) through information-dependent acquisition (IDA) followed by sequential windowed data independent acquisition of the total high-resolution mass spectra (SWATH). The BLASTP was used to perform a local analysis using the predicted Transdecode ORFs from the sialotrancriptome as protein database and the respective peptides from proteomic analysis as a query (E value cutoff of 0.0001, word-size of 7 for a shorter input sequence).

## 4. Protein Structure and Epitope Exposure

The presence of coiled-coil (C), alpha helix (H), and beta sheet (E) was predicted using the NetSurfP-2.0 [75] and BepiPred 2.0 (structural frame) [77] programs. The Chou and Fasman prediction method [78] (from IEDB Analysis Resource, v. 2.22, http://tools.iedb.org/bcell/, accessed on 30 October 2019) was used to predict beta turns within the amino acid sequence, considering probable turn regions those with values higher than 1.

The surface accessibility of the amino acids was evaluated using the Emini prediction method [78] (from IEDB Analysis Resource, v. 2.22, threshold of 1, http://tools.iedb.org/bcell/, accessed on 30 October 2019) and BepiPred 2.0 (surface frame) program. Features such as flexibility and hydrophobicity were evaluated using the Karplus–Schultz [79] and Parker [80] methods (from IEDB Analysis Resource, v. 2.22, threshold of 1, http://tools.iedb.org/bcell/, accessed on 30 October 2019), respectively.

## 5. Prediction of B and T Cell Epitopes

Potential immunogenetic epitopes were predicted using linear B-cell epitope predictors: the Kolaskar and Tongaonkar method [81] (from IEDB Analysis Resource, v. 2.22, threshold of 1, http://tools.iedb.org/bcell/, accessed on 30 October 2019), the predicting antigenic peptides online server (from Immunomedicine Group, http://imed.med.ucm.es/Tools/antigenic.pl, accessed on 30 October 2019), BepiPred 1.0 [80] (from DTU Bioinformatics, http://www.cbs.dtu.dk/services/BepiPred-1.0/, accessed on 30 October 2019), and BepiPred 2.0 (epitope frame) program.

T-cell epitopes were also predicted using all prediction method versions. MHC-I Binding Predictions program (from IEDB Analysis Resource, v. 2.22, http://tools.iedb.org/mhci/, accessed on 30 October 2019) was used to predict epitopes with high affinity to human, mouse, and rat MHC-I. Prediction of MHC-II binding epitopes was obtained using the MHC-II Binding Predictions (from IEDB Analysis Resource, v. 2.22, using all method versions, selecting 12-18-mer peptides, http://tools.iedb.org/mhcii/, accessed on 30 October 2019) and the MHC2Pred (http://crdd.osdd.net/raghava/mhc2pred/, accessed on 30 October 2019) programs for human and mouse MHC-II databases. Predicted T-cell epitopes containing a percentile rank lower or equal to one were selected to identify the representative epitopes using the Epitope Cluster Analysis tools (from IEDB Analysis Resource, v. 2.22, http://tools.iedb.org/cluster/, accessed on 30 October 2019). NetChop (v. 3.1) [82] and PCPS (http://imed.med.ucm.es/Tools/pcps/index.html, accessed on 30 October 2019) programs were used to explore if, after proteasomal processing, the epitope generated could be an MHC binder, which means that could be presented in the host immune system, processed, and ultimately induced in the host humoral and cellular immune pathway.

## 6. Peptide Properties

Amino acid sequence of selected peptides was used to predict it physicochemical characteristics including molecular weight (Da), theoretical isoelectric point (pI), instability index, grand average of hydropathicity (GRAVY), and aliphatic index, using Expasy ProtParam server (http://expasy.org/cgi-bin/protpraram, accessed on 30 November 2019). The allergenicity of the epitopes was predicted by the online servers AllergenFP (v. 1.0) [83], AllerTop (v. 2.0) [84], and AllerCatPro (v. 1.7) [85]. Protein-Sol was used to predict the peptide solubility (>0.8 values indicate a soluble molecule) [86] and CRYSTALP2 for crystallization propensity [87]. Post-translational modification sites in the peptides were predicted using ModPred [88] and PROSITE [89], regarding its impact on protein/peptide production, structure and function [90]. The hemolytic, anti-angiogenic or toxic properties of the selected peptides were analyzed using HemoPI (all SVM methods were used; SVM scores ranges between 0 and 1, i.e., 1 very likely to be hemolytic, 0 very unlikely to be hemolytic) [91], AntiAngioPred (NT15 AAC and whole peptide AAC prediction methods were used; threshold -0,2,) [92], and ToxinPred (all SVM methods were used; E value 10; threshold 0.0) [93], respectively. For comparison, all these analysis were performed for the published synthetic multi-epitope peptide SBm7462^®^ [94]), which has demonstrated to be a protective candidate for a next generation anti-tick vaccine [94,95].

## 7. Results and Discussion

Before searching for promising antigens, the previously published Sequence Read Archives regarding uninfected-unfed, uninfected-fed, and *B. ovis* infected-fed *R. bursa* salivary glands RNA sequencing [59] were reassembled and assessed for completeness using BUSCO analysis [62] and a reference database of 1066 conserved arthropod genes (see Table 1).

The assembly of the sequencing reads of the salivary glands of *B. ovis*-infected-fed *R. bursa* ticks yielded a transcriptome with 70,535 scaffolds, a total assembly size of 64.5 Mbp, and a scaffold N50 length of 1856 bp. The BUSCO completeness report of the assembly indicated that 89.7% complete BUSCOs were obtained for this assembly. Specifically, there were 956 complete (683 complete and single-copy; 273 duplicated), 78 fragmented, and 32 missing BUSCOs. Similarly, a final percentage of complete orthologous genes of 94.0% (uninfected -fed) and 83.7% (uninfected-unfed), respectively, were determined for the remaining uninfected *R. bursa* sialotranscriptomes. It is unlikely to produce a complete BUSCO transcriptome and it is accepted for non-model organisms, such as ticks, to obtain complete scores ranging from 50% to 90% [96]. This is an indicator of a proper transcriptome assembly; thus, these are acceptable ranges for percentage of completeness relative to other RNAseq assemblies in the field [43,97]. Reassembled and complete transcriptomes were considered for the next phase.

### 7.1. Feeding and Pathogen Transmission: Selection of Targets

The systematic workflow of an RV approach must focus on filtering ideal antigens that provide a robust, long-lasting, and deliverable immune response, such as the humoral response, which ultimately interferes with the host-vector-pathogen triad [20]. Thus, the features of an ideal antigen for anti-tick vaccines includes: being a pivotal molecule on tick/pathogen biology, not being homologous to the mammalian host, encoded by a single gene, expressed across life stages and tick tissues, and capable of inducing B and T cells to incite an immunological response without allergenic, hemolytic, and toxic effects [14,98]. Such humoral response is linked to topological features, such as extracellular or intramembrane location, and the presence of coincident epitope groups (CEGs) (also known as “immunological kernels”), are accessible protein regions containing overlapped B and T cell epitopes with ideal chemo-physical properties [52,99,100].

Therefore, the dataset from fed-infected tick salivary glands were analyzed. They were found to correspond to proteins involved in blood feeding and parasite transmission. These processes are intrinsically related to vector survival and competence [101]. Moreover, we performed in silico screening for the antigenic surfaceome and secretome using different filters (Figure 1).

Transcripts containing membrane-related regions were filtered and analyzed regarding its protein antigenicity, homology to vertebrate hosts, cell localization, and annotated function (Supplementary material—Spreadsheet S1). From the transcriptomic selected dataset, 8692 sequences were predicted to be membrane-related proteins that were associated with 5706 different protein family clusters. Each representative of every protein family was investigated for its predicted antigenicity and 1125 proteins were found to be probable antigens in comparison to the Bm86 tick vaccine antigen (Vaxijen score ≥ 0.7). From these predicted antigenic proteins, 859 presented no homology to the vertebrate hosts, warranting the probability of inducing a target tick-specific immune response in the host animal and not leading to an auto-immune phenomenon [25,30,52]. In total, 16 proteins were predicted to be localized in the cell plasma membrane, as a transmembrane protein without signal peptide or GPI anchor (i.e., secreted or anchored). Following this analysis, a putative lipid raft-associated protein containing a MARVEL domain (M_MARVEL, DN25304, EEC06674.1) was identified.

Transcripts containing signal peptides were identified in a similar way as previously described for the transmembrane-related proteins (Supplementary material—Spreadsheet S2). From the current dataset, 4274 sequences contained signal peptides clustered into 3025 different protein families. Predicted antigenicity via Vaxijen identified 623 proteins that presented higher probability of being antigenic relative to Bm86. Alignment and homology analysis indicated that 478 proteins to be tick-specific and non-related to vertebrate hosts (Figure 1). About 200 transcripts were predicted to be extracellular with no predicted membrane-spanning regions following the signal peptide region. Finally, seven putative proteins were identified consisting of two glycine-rich proteins (DN21364, DN28608), an evasin (S_EVASIN, DN20966, AST14849.1), a ricin (S_RICIN, DN33470, EEC03321.1), an antimicrobial peptide (DN7637), and two proteins related to heterodimerization interface (DN16497) and coagulation (DN45898).

### 7.2. In Silico Characterization of Selected Candidates

In this study, one membrane-related (MARVEL) and two secreted (EVASIN, RICIN) proteins were selected to proceed for specific immunoinformatic analysis, since their putative function and occurrence in previously published proteomic data highlighted them as promising targets for anti-tick or disease transmission blocking vaccine development. While MARVEL can be found in the infected-fed, uninfected-fed, and uninfected-unfed conditions of *R. bursa* sialoproteomic data, EVASIN and RICIN are only found in the infected-fed state. This suggests the persistence of MARVEL in the tick cellular machinery as a static membrane protein, while EVASIN and RICIN could be strongly or exclusively linked to infection and feeding.

For each target, prediction methods were used to assess protein structure (Table 2) and putative epitope exposure for a better identification of exposed and immunogenic regions, including B and T (Supplementary material—Spreadsheet S3–S5) cell epitopes. Since very limited information on MHC alleles from sheep and other host vertebrates of *R. bursa* ticks is currently available, “pan-computational methods” predictions were used, as described before [102]. The available allelic datasets from different but well-known hosts, such as humans, mice, and rats, were used to extrapolate the vertebrate host with unknown alleles such as sheep.

Regions with overlapping CEGs were thoroughly examined to screen ideal features for efficient production, using bioinformatic tools to evaluate physicochemical characteristics, post-translational modification sites, propensity for solubility and crystallization, allergenicity, and toxicity (Table 3). Depending on these characteristics, the production and solubilization of these peptides or proteins are different. Finally, as far as possible, the potential negative effects on the host should be predicted *a priori* to testing a potential vaccine [103]. Many highly reactive proteins identified from various parasites (including mites and helminths) are prone to cause allergic reactions in the host [14,104]. Therefore, predicting allergenicity and anti-angiogenic or toxic properties of the selected proteins/peptides are also required when screening for a promising candidate [13]. Taking all the aforementioned components into account, the three chosen candidates are discussed in the following sections.

### 7.3. Putative MARVEL Domain-Containing Protein

**Figure 2:**
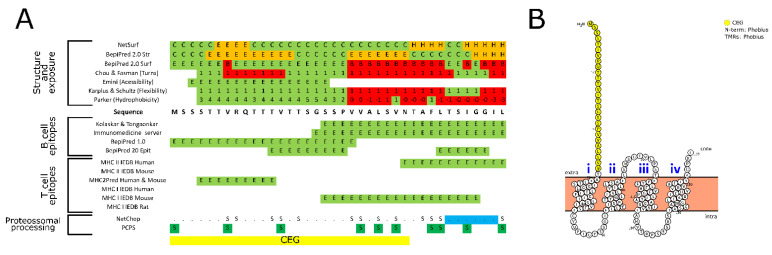
In silico analysis of the putative transmembrane MARVEL protein. (**A**) Identification of overlapping coincident epitopes groups (CEG) using different immunoinformatic approaches. (**B**) Topology prediction (based on Phobious) and localization of the CEG region (yellow) in the protein structure. For detailed information, see the Supplementary material—Spreadsheet S4 and S5.

MARVEL domain-containing proteins generally present an M-shaped topology (four transmembrane-helix region architecture with cytoplasmic N- and C-terminal regions) and function in cholesterol-rich membrane apposition events, such as biogenesis of vesicular transport carriers or tight junction regulation [105]. The putative MARVEL protein identified in this study is 155 amino acids long, thus lacking a signal peptide or GPI-anchor and containing four transmembrane and three extracellular domains (Table 2, Figure 2). From the predicted extracellular domains, only an N-terminal region containing 27 amino acids was predicted to be majorly exposed, with a low structural complexity and a single predicted post-translational modification (PTMs) and glycosylation (Figure 2, Supplementary material—Spreadsheet S3). Such PTMs are associated with protein structure, stability, activity, trafficking, and protein–protein interactions [90]. All of these targeted characteristics are being considered for a potential vaccine candidate. The propensity of this protein to induce the humoral pathway was evaluated by predicting in silico the B and T cell epitopes (Figure 2, Supplementary material—Spreadsheet S3). The three B cell epitope predictors showed that this segment of the MARVEL protein could be presented directly to B cells and induce a humoral response. Additionally, this protein portion has predicted protease cut sites (pos. 32-37) that could originate peptides that would enable presentation through MHC I (pos. 18-27) and II (pos. 4-12) (Figure 2, Supplementary material—Spreadsheet S3).

The predicted coincident epitope (MSSSTTVRQTTTVTTSGSSPVVALSVN) possesses flexibility and hydrophobicity which makes this fragment a promising candidate for synthetic production (Figure 2, Supplementary material—Spreadsheet S3). Other predictions were performed (Table 3), which indicated that the peptide alone is alkaline with a high probability to be unstable and hydrophobic. Nevertheless, peptide bioengineering by selection of a compatible carrier protein or linkage to other promising targets may contribute towards alleviating such drawbacks.

This peptide has more thermostability and solubility than Bm86-derived peptide. It also has low probability in causing allergic host reactions and hemolysis. Further, it can be anti-angiogenic or toxic. These are all characteristics that improve the use of this antigen for vaccine administration.

### 7.4. Putative Evasin

Other studies have mentioned that evasins are a secreted salivary glycoprotein that enables the endurance of tick feeding by suppressing the host immune response [106]. During blood feeding, such molecules are injected into the tick bite site and bind to the host chemokines to inhibit its function, resulting in a prevention of chemotaxis of leukocytes and subverting the host anti-inflammatory immune response associated to this phenomenon [106]. Besides, evasins can be ubiquitously expressed by a wide variety of tick species, constituting a promising target as an anti-tick vaccine that needs to be explored [48,106].

**Figure 3:**
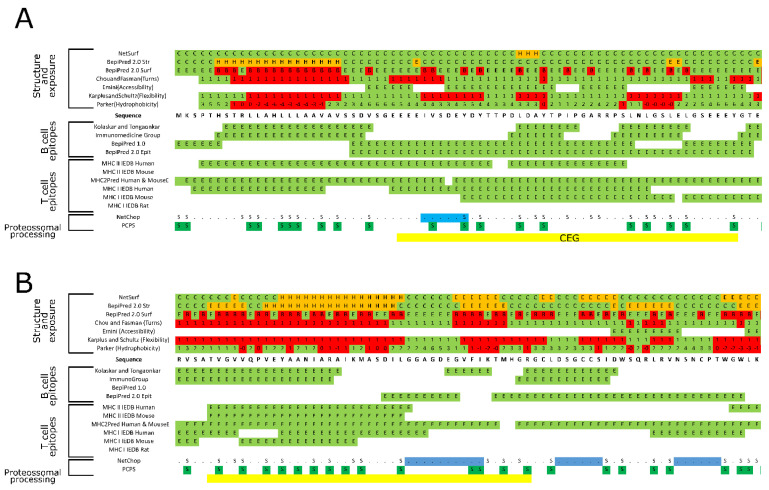
In silico analysis of the two putative signal proteins: EVASIN and RICIN. Identification of overlapping coincident epitopes groups (CEGs) within (**A**) EVASIN and (**B**) RICIN amino acids sequences. For detailed information see Supplementary material—Spreadsheet S4 and S5.

The putative evasin identified in this study contained an N-terminal signal peptide and no transmembrane helices (Table 2), suggesting that it might be a secretory protein. Moreover, this sequence has high homology to an evasin protein from *Rhipicephalus microplus* (AST14849) and possess several characteristics from the evasin protein family [106], such as nine Cys residues and N-linked glycosylation, as well as putative tyrosine sulfation sites (Figure 3A, Supplementary material—Spreadsheet S4). A region following the predicted signal peptide (between pos. 29 and 70) has a low complexity structure with several exposed residues (Figure 3A, Supplementary material—Spreadsheet S4), which could facilitate epitope presentation. Predictors indicates that the protein could be potentially cleaved in some positions, e.g., pos. 26 to 36 and 98 to 103, leaving a peptide portion (37–97) to be potentially processed and presented by the MHCs. Within this secretory region, many putative epitopes can be detected and processed by B and T cells containing a few putative PTMs (i.e., phosphorylation and sulfation) (Supplementary material—Spreadsheet S4). In this predicted highly immunogenic region, a peptide (EEEIVSDEYDYTTPDLDAYTPIPGARRPSLNLGSLELGSEEEY, pos. 29 to 71) was selected to be further evaluated in silico (Table 3). Predictions revealed that this peptide is acidic and unstable upon synthesis but such can be surmounted as previously referenced, in order to benefit on the other properties, such as hydrophilicity, solubility, and having no negative impacts on the host.

### 7.5. Putative Ricin

The most characteristic though not completely conserved sequence feature of ricin B lectin domains is the presence of a Q-W repeats containing an omega loop but no major segments of a helix or beta sheet throughout the sequence [107,108]. The primary structure of ricin proteins has shown the presence of a similar domain in many carbohydrate-recognition proteins like plant, fungi, and bacteria AB-toxins, glycosidases, or proteases [107,108,109]. Proteins containing such domains are linked to cytotoxicity [110,111], cytoadhering [112], and possess immunomodulatory properties [113,114,115]. From the dataset, one sequence shows similarity to a ricin B lectin domain (Table 2), but no Q-W repeats were found. The 133 amino acid sequence contains a signal peptide and no transmembrane helices (Table 2), indicating that it might be secreted. Myristoylation and phosphorylation PTMs were predicted in this sequence (Supplementary material—Spreadsheet S5). Even with a complex structure with alpha helixes and beta sheets that reduces the exposure of epitopes, this sequence has regions that are likely to be recognized by B cell receptors, as well as MHC I and MHC II receptors of different organisms (Figure 3B, Supplementary material—Spreadsheet S5). We identified an N-terminal peptide region (TVGVVQPVEYAANIARAIKMASDILGGAGDEGVFIKTMHGR) that possesses more predicted B and T cell epitopes than the remaining sequence and flanked by an enzymatic cleavage site. Most of the predictors indicated that even with some undesirable characteristics (such as instability index and GRAVY), this peptide has a pI closer to seven as the SBm7462^®^ peptide, could be thermostable, soluble, and present no harmful properties to the host (Table 3).

## 8. Conclusions and Future Perspectives

Transmission-blocking vaccines are considered essential tools for interrupting disease transmission. An immunized host produces inhibitory antibodies against pathogen/vector antigens that are ingested by the vector during blood feeding, interfering ultimately with vector competence and disease transmission [18]. The cellular pathway is compromised since the antibodies alter the activity or signal transduction of proteins through a physical block [116,117]. The discovery of new antigens is a prerequisite in developing new diagnostics and vaccines for disease surveillance and control. Reverse vaccinology is a preferable approach to overcome the time and resources required to obtain promising candidates. However, there is an urgent need to develop a pipeline to run multiple algorithms in a single platform focused on tick research, including information on tick omics data, vertebrate hosts immune databases (from livestock, domestic animals, humans, including information about the epitope repertoire, and broad population coverage), and proteins from transmitted pathogens.

This study combines the power of several bioinformatics tools to establish a rational pipeline for vaccine antigen discovery. Focusing on peptide design will greatly reduce the cost of a putative vaccine and enhance its accessibility to the community, since smaller biomolecules are easier to synthetize and store [118]. Thus, three peptides that showed the desired characteristics were identified for further testing as next-generation vaccine targets.

These promising antigens will be tested in a follow-up study, yet prior to vaccination trials, a thorough investigation should be conducted to survey the humoral immune response by animals from tick endemic regions to these peptides. A preexistent humoral response to the antigens identified here, within an endemic area, will demonstrate that such molecules do not protect the host from tick infestation. Alternatively, an absence of an established and natural humoral response to these peptides might lend weight their use in a new protective strategy or even as diagnostic markers. Interestingly, the evaluation of the expression of such targets in cells, different tissues/fluids, and developmental stages of *R. bursa* tick species, could elucidate their applicability as broad-spectrum tick antigens. Several approaches to elucidate or validate the in vivo cellular localization (e.g., immunofluorescence and western-blot assays), protein structure (e.g., crystallography), and protein–protein interactions (e.g., yeast two-hybrid, etc.) could be conducted in future for top selected predicted targets. Pilot vaccination trials are needed to in vivo validate the immunogenicity of peptides where different aspects should be taken in account, such as antigen design/production (peptide, native protein, synthetic, polymers, type of host expression system, recombination with other promising antigens, linkers, etc.), its administration (route/system, dose, adjuvant), the host response (humoral and cellular immune response, physiological and clinical responses), and the influence on tick behavior and physiological features.

## Figures and Tables

**Table 1:** BUSCO statistics for each *Rhipicephalus bursa* sialotranscriptome assembly against an arthropod database. Conserved BUSCO genes were assigned to four classes of genes: missing, fragmented, duplicated, and complete.

Dataset	*Babesia ovis* Infected and Fed	Uninfected and Fed	Uninfected and Unfed
Scaffolds (#)	70535	63942	58670
Assembly size (Mbp)	64.5	67.6	47.3
N50 (bp)	1856	2266	1522
Number of conserved arthropod genes in BUSCO reference set	1066		
Complete and single-copy	683 (64.1%)	690 (64.7%)	692 (64.9%)
Complete and duplicated	273 (25.6%)	312 (29.3%)	200 (18.8%)
Fragmented	78 (7.3%)	36 (3.4%)	125 (11.7%)
Missing	32 (3%)	28 (2.6%)	49 (4.6%)

**Table 2:** Topology and structure properties of the selected targets. Several bioinformatic tools were used to obtain this data. (Prosite (*), VectorBase (**), Phobius (a), TMHMM (b), CCTOP (c), SACS TMHMM (d), and SignalP (e).)

Protein Name	Length (aa)	MW (Da)	pI	Functional Domains	Transmembrane Domains	Extracellular Domains	SP	GPI
MARVEL	155	16508.53	9.03	Contains: leucine zipper domain (pos. 24-52) * andMarvel domain (pos. 29-157 from ISCW003585) **	Pos. 29-52, 64-84, 96-117, 129-150 a Pos. 28-50, 63-85, 98-120, 127-149 b Pos. 29-52, 62-85, 95-117, 127-149 c Pos. 28-50, 63-85, 98-120, 127-149 d	Pos. 1-28, 85-95, 151-155 a Pos. 1-27, 86-97, 150-155 b Pos. 1-28, 86-94, 150-155 c Pos. 1-27, 86-97, 150-155 d	No	No
EVASIN	164	17681.84	4.20	Homology to an evasin protein (AST14849)	None	Pos. 28-164 a Pos. 1-164 b Pos. 29-164 c	Yes Pos. 1-27 a Pos. 1-26 e	No
RICIN	133	14401.49	8.19	Homology to a hypothetical protein which contains a Ricin-type beta-trefoil lectin domain (EEC03321)	None	Pos. 1-133 a Pos. 1-133 b Pos. 40-133 c	Yes Pos. 1-39 c Pos. 1-37 e	No

**Table 3:** Physicochemical properties of the selected overlapping coincident epitopes groups (CEGs) from MARVEL, EVASIN, and RICIN. “+” represents high probability, “-“ represents low probability.

Protein Name	CEG Length (aa)	Molecular Weight (Da)	pI	Instability Index	GRAVY	Aliphatic Index	Allergenicity (AllerFP/AllerTop/AllerCatPro)	Solubility and Crystallization Propensity (Protein-Sol, CRYSTALP2)	Hemolytic Potency (HemoPI)	Anti-Angiogenic Property (AntiAngioPred)	Toxicity Prediction (ToxinPred)
MARVEL	27	2698.98	9.5	74.70	0.167	71.85	-/+/-	0.669, none	0.01, 0.49, 0.44, 0.00, 0.49	+, +	-, -, -, -
EVASIN	43	4835.13	3.69	66.52	-0.856	74.88	-/-/-	0.662, none	0.00, 0.47, 0.33, 0.00, 0.47	-, -	-, -, -, -
RICIN	41	4243.91	6.43	57.47	0.285	97.56	-/+/-	0.659, none	0.00, 0.48, 0.47, 0.00, 0.48	-, -	-, -, -, -
SBm7462^®^	45	5056.78	6.87	49.21	-0.300	47.78	-/-/-	0.484, none	0.45, 0.35, 0.37, 0.00, 0.35	+, +	+, -, +, -

## Data Availability

The data presented in this study is available as supplementary material that can be found at www.mdpi.com/xxx/s1.

## Supplementary Materials

The following are available online at https://www.mdpi.com/article/10.3390/biomedicines9040363/s1, Spreadsheet S1: Screening of assembled transcripts containing membrane-related regions, regarding protein antigenicity, homology to vertebrate hosts, cell localization, and annotated function. Spreadsheet S2: Screening of assembled transcripts containing signal peptides, regarding protein antigenicity, homology to vertebrate hosts, cell localization, and annotated function. Spreadsheet S3: Detailed information regarding putative MARVEL in silico characterization, including protein structure and putative epitope exposure (B and T cell epitopes). Spreadsheet S4: Detailed information regarding putative EVASIN in silico characterization, including protein structure, putative epitope exposure (B and T cell epitopes). Spreadsheet S5: Detailed information regarding putative RICIN in silico characterization, including protein structure, putative epitope exposure (B and T cell epitopes).
